# [Bis­(4-methyl-1,3-thia­zol-2-yl-κ*N*)methane]­tricarbonyl­dichlorido­tungsten(II)

**DOI:** 10.1107/S1600536811038104

**Published:** 2011-09-30

**Authors:** Christoph E. Strasser, Stephanie Cronje, Helgard G. Raubenheimer

**Affiliations:** a Department of Chemistry and Polymer Science, University of Stellenbosch, Private Bag X1, Matieland, 7602, South Africa

## Abstract

The title compound, [WCl_2_(C_9_H_10_N_2_S_2_)(CO)_3_], is a hepta­coordinate tungsten(II) complex with a capped–octa­hedral coordination sphere in which one CO ligand caps a face formed by a chloro ligand and the two other carbonyls. The chloro ligands are mutually *trans* positioned at an angle of 156.98 (7)°. The chelating bis­(4-methyl-1,3-thia­zol-2-yl)methane ligand coordinates with the imine N atoms. In the crystal, mol­ecules are linked into chains parallel to [201] by weak C—H⋯O contacts between the CH_2_ group of the bis­(4-methyl­thia­zol-2-yl)methane ligand and the O atom of the capping CO group.

## Related literature

For related compounds, see: Baker *et al.* (1986 ▶); Moss & Smith (1983 ▶); Stiddard (1962 ▶); Szymanska-Buzar (1989 ▶); Tripathi *et al.* (1976 ▶). For related structures, see: Baker *et al.* (1996 ▶, 2000 ▶); Drew *et al.* (1988 ▶, 1995 ▶); Hillhouse *et al.* (1982 ▶); Shiu *et al.* (1990 ▶). For the isolation of the title compound, see: Strasser *et al.* (2009 ▶).
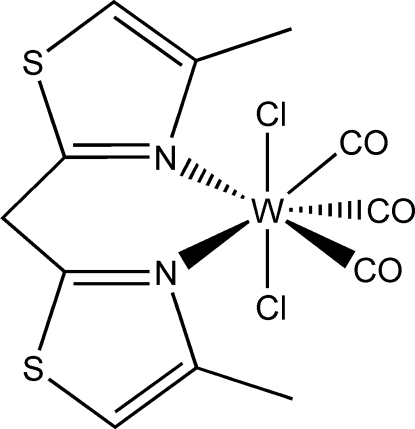


## Experimental

### 

#### Crystal data

[WCl_2_(C_9_H_10_N_2_S_2_)(CO)_3_]
*M* *_r_* = 549.10
Monoclinic, 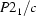

*a* = 8.6876 (17) Å
*b* = 12.912 (2) Å
*c* = 14.851 (3) Å
β = 105.550 (3)°
*V* = 1604.9 (5) Å^3^
*Z* = 4
Mo *K*α radiation
μ = 7.80 mm^−1^
*T* = 100 K
0.13 × 0.13 × 0.04 mm

#### Data collection

Bruker APEX CCD diffractometer
Absorption correction: multi-scan (*SADABS*; Bruker, 2002 ▶) *T*_min_ = 0.549, *T*_max_ = 0.772
9133 measured reflections
3310 independent reflections
2843 reflections with *I* > 2σ(*I*)
*R*_int_ = 0.037

#### Refinement

*R*[*F* ^2^ > 2σ(*F* ^2^)] = 0.042
*wR*(*F* ^2^) = 0.099
*S* = 1.07
3310 reflections
201 parameters
H-atom parameters constrained
Δρ_max_ = 3.92 e Å^−3^
Δρ_min_ = −2.06 e Å^−3^

### 

Data collection: *SMART* (Bruker, 2002 ▶); cell refinement: *SAINT* (Bruker, 2003 ▶); data reduction: *SAINT*; program(s) used to solve structure: *SHELXS97* (Sheldrick, 2008 ▶); program(s) used to refine structure: *SHELXL97* (Sheldrick, 2008 ▶); molecular graphics: *X-SEED* (Barbour, 2001 ▶; Atwood & Barbour, 2003 ▶); software used to prepare material for publication: *X-SEED*.

## Supplementary Material

Crystal structure: contains datablock(s) I, Global. DOI: 10.1107/S1600536811038104/rk2300sup1.cif

Structure factors: contains datablock(s) I. DOI: 10.1107/S1600536811038104/rk2300Isup2.hkl

Additional supplementary materials:  crystallographic information; 3D view; checkCIF report

## Figures and Tables

**Table 1:** Hydrogen-bond geometry (Å, °)

*D*—H⋯*A*	*D*—H	H⋯*A*	*D*⋯*A*	*D*—H⋯*A*
C10—H10*A*⋯O2^i^	0.99	2.38	3.28 (1)	151

Symmetry code: (i) 
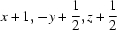
.

**Fig. 1.**
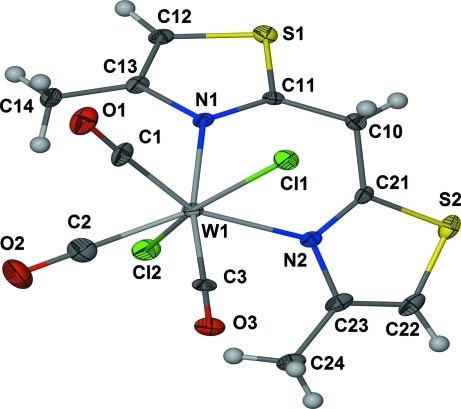
Molecular structure of the title compound with the atom numbering scheme. Displacement ellipsoids are drawn at the 50% probability level. Hydrogen atoms are shown as spheres of arbitrary radius.

## supplementary crystallographic information

### Comment

Heptacoordinate W(II) complexes are common due to the 18–electron configuration at the metal centre. The title compound, shown in Fig. 1, was obtained through unclear side reactions which involve the formation of *bis*(4–methyl–1,3–thiazol–2–yl)methane from the anionic (4–methyl–1,3–thiazol–2–yl)carbonyl or activated (4–methyl–1,3–thiazol–2-yl)(trichloromethoxycarbonyl)methylene ligands as well as concomitant oxidation of W(0) to W(II).

Complexes of the type [W*X*_2_(CO)_3_(*L*)_2_] (*X* = Cl, Br or I; *L* = *N*–donor ligand) have been synthesized by photochemical reaction of *e.g.* [W(CO)_6_], CCl_4_ and 2,2'–bipyridine (*bipy*) to yield [WCl_2_(CO)_3_(*bipy*)] (Szymanska-Buzar, 1989), oxidation of [W(CO)_4_(*L*)_2_] (Stiddard *et al.*, 1962) or [W(CO)_3_(CH_3_CN)_3_] (Baker *et al.*, 1986) with bromine or iodine or reaction of [W*X*_3_(CO)_4_]^-^ (*X* = Br or I) with *bipy* (Moss & Smith, 1983).

This is the first structural determination of a [W*X*_2_(CO)_3_(*L*)_2_]–type complex with chloro ligands. Such complexes (*X* = Cl) with monodentate *L* = nitriles (Baker *et al.*, 1986) and *L* = alkylamines (Tripathi *et al.*, 1976) were reported to be highly unstable. It is therefore surprising that for the present compound no decomposition, *e.g.* decarbonylation (Shiu *et al.*, 1990) was encountered when crystals were briefly exposed to oxygen, room temperature and light during set–up of the X–ray diffraction experiment. The chelating *bis*(4–methylthiazol–2–yl)methane ligand may exert additional stabilizing properties when compared to the ligands used in the literature.

Crystal and molecular structures of seven–coordinate complexes of the type [W*X*_2_(CO)_3_(*R*CN)_2_] (*R*CN is an organic nitrile) have been reported by Baker *et al.* (1986, 1996, 2000) and Drew *et al.* (1988, 1995). The W—N bond distances in these nitrile complexes are shorter than those found in the title compound while other geometrical parameters are similar. The nitrile complexes also exhibit capped–octahedral geometry with *trans*–disposed iodo ligands. They possess a mirror plane that bisects the molecule while in the title compound the whole molecule is asymmetric; the position of the carbonyl ligands with respect to the bidentate *bis*(thiazolyl)methane is incompatible with *C*_s_ symmetry. Hillhouse *et al.* (1982) report coordination of a tetraarylphosphazide (*Ph*NNNP*Ph*_3_) to a dibromotricarbonyltungsten fragment which is different from the title compound and the structures mentioned here in that it contains a set of *cis*–bromo ligands, possibly caused by the smaller bite angle of the tetraarylphosphazide (N—W—N angle of 56.7 (2)° as opposed to the N1—W1—N2 angle measuring 83.3 (2)° in the title compound). Finally, a geometrically very similar complex to the one reported here but utilizing a *bis*(azolyl)methane ligand was prepared by Shiu *et al.*, (1990) [WBr_2_(CO)_3_(CH_2_*R*_2_)] (*R* = 3,4,5–trimethyl–1*H*–pyrazol–1–yl–κ*N*^2^).

The significantly longer W1—Cl2 bond (2.528 (2)Å) in the title compound is adjacent to the capping CO ligand while the W1—Cl1 bond is undisturbed by a capping ligand and measures 2.4708 (17)Å. The same effect is observed to a variable degree in all structures mentioned here for comparison. The individual molecules of the title compound are arranged into chains parallel to the [2 0 1] line by weak C—H···O contacts between the CH_2_ group of the bis(4–methylthiazol–2–yl)methane ligand and O2 of the capping CO group.

### Experimental

A crystal of the tile compound was isolated when tetramethylammonium pentacarbonyl[(4–methyl–1,3–thiazol–5–yl)carbonyl]tungstate(1-) was treated with bis(trichloromethyl)carbonate and pyridine to obtain the carbyne complex [W(≡CC_4_H_4_NS)Cl(CO)_2_(py)_2_] by oxide abstraction (Strasser *et al.*, 2009). Decomposition concomitant with development of a green colour was noticed; the reaction mixture was chromatographed on Florisil at 243 K using CH_2_Cl_2_/acetonitrile mixtures and an yellow fraction was obtained containing the title compound which was crystallized from CH_2_Cl_2_/pentane at 253 K.

### Refinement

All H atoms were positioned geometrically (C—H = 0.95Å, 0.99Å and 0.98Å for CH, CH_2_ and CH_3_ groups, respectively) and constrained to ride on their parent atoms; *U*_iso_(H) values were set at 1.2*U*_eq_(C) for CH– and CH_2_–groups and 1.5*U*_eq_(C) for CH_3_–groups.

The maximum residual electron density of 3.92 e×Å^-3^ is located 0.79Å near W1.

### Crystal data

**Table:**

[WCl_2_(C_9_H_10_N_2_S_2_)(CO)_3_]	*F*(000) = 1040
*M**_r_* = 549.10	*D*_x_ = 2.273 Mg m^−^^3^
Monoclinic, *P*2_1_/*c*	Mo *K*α radiation, λ = 0.71073 Å
Hall symbol: -P 2ybc	Cell parameters from 2900 reflections
*a* = 8.6876 (17) Å	θ = 2.9–26.4°
*b* = 12.912 (2) Å	µ = 7.80 mm^−^^1^
*c* = 14.851 (3) Å	*T* = 100 K
β = 105.550 (3)°	Prism, yellow
*V* = 1604.9 (5) Å^3^	0.13 × 0.13 × 0.04 mm
*Z* = 4	

### Data collection

**Table:**

Bruker APEX CCD diffractometer	3310 independent reflections
Radiation source: fine–focus sealed tube	2843 reflections with *I* > 2σ(*I*)
graphite	*R*_int_ = 0.037
ω–scans	θ_max_ = 26.5°, θ_min_ = 2.1°
Absorption correction: multi-scan (*SADABS*; Bruker, 2002)	*h* = −9→10
*T*_min_ = 0.549, *T*_max_ = 0.772	*k* = −14→16
9133 measured reflections	*l* = −18→16

### Refinement

**Table:**

Refinement on *F*^2^	Primary atom site location: structure-invariant direct methods
Least-squares matrix: full	Secondary atom site location: difference Fourier map
*R*[*F*^2^ > 2σ(*F*^2^)] = 0.042	Hydrogen site location: inferred from neighbouring sites
*wR*(*F*^2^) = 0.099	H-atom parameters constrained
*S* = 1.07	*w* = 1/[σ^2^(*F*_o_^2^) + (0.041*P*)^2^ + 16.6008*P*] where *P* = (*F*_o_^2^ + 2*F*_c_^2^)/3
3310 reflections	(Δ/σ)_max_ = 0.001
201 parameters	Δρ_max_ = 3.92 e Å^−^^3^
0 restraints	Δρ_min_ = −2.06 e Å^−^^3^

### Special details

**Table:**

Geometry. All s.u.'s (except the s.u. in the dihedral angle between two l.s. planes) are estimated using the full covariance matrix. The cell s.u.'s are taken into account individually in the estimation of s.u.'s in distances, angles and torsion angles; correlations between s.u.'s in cell parameters are only used when they are defined by crystal symmetry. An approximate (isotropic) treatment of cell s.u.'s is used for estimating s.u.'s involving l.s. planes.
Refinement. Refinement of *F*^2^ against ALL reflections. The weighted *R*–factor *wR* and goodness of fit *S* are based on *F*^2^, conventional *R*–factors *R* are based on *F*, with *F* set to zero for negative *F*^2^. The threshold expression of *F*^2^ > 2σ(*F*^2^) is used only for calculating *R*–factors(gt) *etc*. and is not relevant to the choice of reflections for refinement. *R*–factors based on *F*^2^ are statistically about twice as large as those based on *F*, and *R*–factors based on ALL data will be even larger.

### Fractional atomic coordinates and isotropic or equivalent isotropic displacement parameters (Å^2^)

**Table:**

	*x*	*y*	*z*	*U*_iso_*/*U*_eq_	
W1	0.71470 (4)	0.28785 (2)	0.11943 (2)	0.01491 (11)	
Cl1	0.9818 (2)	0.29333 (15)	0.09055 (14)	0.0206 (4)	
S1	0.9434 (2)	0.05707 (15)	0.36702 (13)	0.0172 (4)	
O1	0.7129 (7)	0.1221 (5)	−0.0344 (4)	0.0299 (15)	
N1	0.7939 (7)	0.1559 (5)	0.2219 (4)	0.0133 (13)	
C1	0.7098 (10)	0.1829 (7)	0.0212 (6)	0.0224 (18)	
Cl2	0.5130 (2)	0.28940 (16)	0.21453 (15)	0.0252 (4)	
S2	1.0291 (3)	0.44059 (16)	0.39914 (14)	0.0211 (4)	
O2	0.3687 (8)	0.2745 (6)	−0.0173 (5)	0.0399 (17)	
N2	0.8330 (7)	0.3872 (5)	0.2453 (4)	0.0133 (13)	
C2	0.4955 (11)	0.2788 (7)	0.0348 (6)	0.0260 (19)	
O3	0.6745 (7)	0.4803 (4)	−0.0140 (4)	0.0226 (13)	
C3	0.6882 (9)	0.4116 (6)	0.0370 (5)	0.0148 (15)	
C10	1.0349 (9)	0.2488 (6)	0.3165 (6)	0.0171 (16)	
H10B	1.1001	0.2476	0.2710	0.020*	
H10A	1.1078	0.2383	0.3796	0.020*	
C11	0.9176 (9)	0.1625 (6)	0.2950 (5)	0.0132 (15)	
C12	0.7790 (9)	−0.0001 (6)	0.2943 (5)	0.0171 (16)	
H12	0.7394	−0.0665	0.3044	0.020*	
C13	0.7126 (9)	0.0615 (6)	0.2203 (5)	0.0169 (16)	
C14	0.5711 (9)	0.0317 (6)	0.1437 (6)	0.0202 (17)	
H14A	0.6049	0.0153	0.0874	0.030*	
H14B	0.4949	0.0894	0.1304	0.030*	
H14C	0.5200	−0.0291	0.1626	0.030*	
C21	0.9561 (9)	0.3533 (6)	0.3128 (5)	0.0143 (15)	
C22	0.8855 (10)	0.5279 (6)	0.3436 (6)	0.0211 (17)	
H22	0.8738	0.5955	0.3663	0.025*	
C23	0.7924 (10)	0.4884 (6)	0.2641 (6)	0.0182 (16)	
C24	0.6582 (10)	0.5456 (6)	0.1992 (6)	0.0202 (17)	
H24A	0.6305	0.6063	0.2313	0.030*	
H24B	0.5650	0.5000	0.1797	0.030*	
H24C	0.6911	0.5681	0.1441	0.030*	

### Atomic displacement parameters (Å^2^)

**Table:**

	*U*^11^	*U*^22^	*U*^33^	*U*^12^	*U*^13^	*U*^23^
W1	0.01665 (17)	0.01210 (17)	0.01458 (17)	0.00270 (13)	0.00174 (11)	−0.00111 (12)
Cl1	0.0161 (9)	0.0220 (10)	0.0258 (10)	0.0004 (8)	0.0093 (8)	−0.0014 (8)
S1	0.0171 (9)	0.0187 (10)	0.0165 (9)	0.0016 (8)	0.0059 (7)	0.0038 (7)
O1	0.032 (4)	0.031 (4)	0.031 (3)	−0.008 (3)	0.016 (3)	−0.011 (3)
N1	0.014 (3)	0.011 (3)	0.017 (3)	0.001 (2)	0.008 (3)	−0.003 (2)
C1	0.021 (4)	0.019 (4)	0.029 (5)	−0.009 (3)	0.011 (4)	−0.007 (4)
Cl2	0.0244 (10)	0.0221 (10)	0.0326 (11)	−0.0001 (8)	0.0137 (9)	−0.0007 (9)
S2	0.0273 (11)	0.0214 (11)	0.0154 (9)	−0.0090 (8)	0.0070 (8)	−0.0049 (8)
O2	0.024 (4)	0.048 (5)	0.042 (4)	−0.002 (3)	−0.002 (3)	0.010 (3)
N2	0.014 (3)	0.012 (3)	0.016 (3)	0.000 (2)	0.009 (3)	−0.001 (2)
C2	0.030 (5)	0.030 (5)	0.019 (4)	0.000 (4)	0.007 (4)	0.003 (4)
O3	0.024 (3)	0.021 (3)	0.025 (3)	0.006 (2)	0.010 (3)	0.004 (2)
C3	0.016 (4)	0.014 (4)	0.015 (4)	0.008 (3)	0.005 (3)	−0.003 (3)
C10	0.014 (4)	0.016 (4)	0.020 (4)	0.000 (3)	0.003 (3)	0.001 (3)
C11	0.013 (4)	0.012 (4)	0.015 (4)	0.003 (3)	0.004 (3)	0.001 (3)
C12	0.019 (4)	0.014 (4)	0.022 (4)	0.003 (3)	0.011 (3)	−0.002 (3)
C13	0.015 (4)	0.016 (4)	0.024 (4)	0.000 (3)	0.012 (3)	−0.005 (3)
C14	0.017 (4)	0.016 (4)	0.028 (4)	−0.003 (3)	0.007 (3)	−0.005 (3)
C21	0.014 (4)	0.012 (4)	0.019 (4)	−0.004 (3)	0.007 (3)	−0.004 (3)
C22	0.031 (5)	0.014 (4)	0.022 (4)	−0.008 (3)	0.015 (4)	−0.004 (3)
C23	0.027 (4)	0.012 (4)	0.022 (4)	−0.003 (3)	0.016 (3)	0.001 (3)
C24	0.026 (4)	0.015 (4)	0.023 (4)	0.003 (3)	0.013 (3)	−0.001 (3)

### Geometric parameters (Å, °)

**Table:**

W1—C1	1.983 (8)	O3—C3	1.151 (9)
W1—C2	1.984 (9)	C10—C11	1.487 (11)
W1—C3	1.989 (8)	C10—C21	1.506 (11)
W1—N1	2.265 (6)	C10—H10B	0.9900
W1—N2	2.273 (6)	C10—H10A	0.9900
W1—Cl1	2.4708 (19)	C12—C13	1.354 (11)
W1—Cl2	2.528 (2)	C12—H12	0.9500
S1—C11	1.708 (7)	C13—C14	1.484 (11)
S1—C12	1.710 (8)	C14—H14A	0.9800
O1—C1	1.145 (10)	C14—H14B	0.9800
N1—C11	1.310 (9)	C14—H14C	0.9800
N1—C13	1.405 (10)	C22—C23	1.340 (11)
S2—C21	1.698 (7)	C22—H22	0.9500
S2—C22	1.720 (9)	C23—C24	1.493 (11)
O2—C2	1.165 (11)	C24—H24A	0.9800
N2—C21	1.328 (10)	C24—H24B	0.9800
N2—C23	1.402 (10)	C24—H24C	0.9800
			
C1—W1—C2	70.5 (4)	C21—C10—H10B	109.1
C1—W1—C3	96.9 (3)	C11—C10—H10A	109.1
C2—W1—C3	74.0 (3)	C21—C10—H10A	109.1
C1—W1—N1	85.6 (3)	H10B—C10—H10A	107.8
C2—W1—N1	116.6 (3)	N1—C11—C10	126.1 (7)
C3—W1—N1	169.2 (3)	N1—C11—S1	114.1 (6)
C1—W1—N2	155.0 (3)	C10—C11—S1	119.7 (5)
C2—W1—N2	134.4 (3)	C13—C12—S1	111.1 (6)
C3—W1—N2	90.3 (3)	C13—C12—H12	124.4
N1—W1—N2	83.3 (2)	S1—C12—H12	124.4
C1—W1—Cl1	74.2 (2)	C12—C13—N1	113.1 (7)
C2—W1—Cl1	132.7 (2)	C12—C13—C14	123.7 (7)
C3—W1—Cl1	80.2 (2)	N1—C13—C14	123.2 (7)
N1—W1—Cl1	90.47 (16)	C13—C14—H14A	109.5
N2—W1—Cl1	83.58 (16)	C13—C14—H14B	109.5
C1—W1—Cl2	122.3 (2)	H14A—C14—H14B	109.5
C2—W1—Cl2	70.3 (3)	C13—C14—H14C	109.5
C3—W1—Cl2	110.8 (2)	H14A—C14—H14C	109.5
N1—W1—Cl2	76.20 (16)	H14B—C14—H14C	109.5
N2—W1—Cl2	76.36 (16)	N2—C21—C10	126.1 (7)
Cl1—W1—Cl2	156.98 (7)	N2—C21—S2	114.4 (6)
C11—S1—C12	90.1 (4)	C10—C21—S2	119.5 (6)
C11—N1—C13	111.6 (6)	C23—C22—S2	111.2 (6)
C11—N1—W1	122.8 (5)	C23—C22—H22	124.4
C13—N1—W1	125.4 (5)	S2—C22—H22	124.4
O1—C1—W1	177.5 (8)	C22—C23—N2	113.9 (7)
C21—S2—C22	89.8 (4)	C22—C23—C24	124.2 (7)
C21—N2—C23	110.6 (6)	N2—C23—C24	121.8 (7)
C21—N2—W1	122.0 (5)	C23—C24—H24A	109.5
C23—N2—W1	127.4 (5)	C23—C24—H24B	109.5
O2—C2—W1	177.8 (8)	H24A—C24—H24B	109.5
O3—C3—W1	176.7 (6)	C23—C24—H24C	109.5
C11—C10—C21	112.6 (6)	H24A—C24—H24C	109.5
C11—C10—H10B	109.1	H24B—C24—H24C	109.5
			
C1—W1—N1—C11	130.7 (6)	W1—N1—C11—S1	174.8 (3)
C2—W1—N1—C11	−163.7 (6)	C21—C10—C11—N1	51.6 (10)
C3—W1—N1—C11	26.9 (16)	C21—C10—C11—S1	−131.1 (6)
N2—W1—N1—C11	−26.9 (6)	C12—S1—C11—N1	0.5 (6)
Cl1—W1—N1—C11	56.6 (5)	C12—S1—C11—C10	−177.1 (6)
Cl2—W1—N1—C11	−104.4 (6)	C11—S1—C12—C13	−0.1 (6)
C1—W1—N1—C13	−54.4 (6)	S1—C12—C13—N1	−0.2 (8)
C2—W1—N1—C13	11.1 (7)	S1—C12—C13—C14	178.2 (6)
C3—W1—N1—C13	−158.2 (12)	C11—N1—C13—C12	0.6 (9)
N2—W1—N1—C13	148.0 (6)	W1—N1—C13—C12	−174.8 (5)
Cl1—W1—N1—C13	−128.6 (5)	C11—N1—C13—C14	−177.8 (7)
Cl2—W1—N1—C13	70.4 (5)	W1—N1—C13—C14	6.8 (10)
C1—W1—N2—C21	−34.2 (10)	C23—N2—C21—C10	−179.1 (7)
C2—W1—N2—C21	151.0 (6)	W1—N2—C21—C10	0.6 (10)
C3—W1—N2—C21	−141.4 (6)	C23—N2—C21—S2	−1.0 (8)
N1—W1—N2—C21	29.9 (6)	W1—N2—C21—S2	178.7 (3)
Cl1—W1—N2—C21	−61.3 (5)	C11—C10—C21—N2	−47.1 (10)
Cl2—W1—N2—C21	107.3 (6)	C11—C10—C21—S2	134.8 (6)
C1—W1—N2—C23	145.4 (7)	C22—S2—C21—N2	0.8 (6)
C2—W1—N2—C23	−29.4 (8)	C22—S2—C21—C10	179.1 (6)
C3—W1—N2—C23	38.2 (6)	C21—S2—C22—C23	−0.5 (6)
N1—W1—N2—C23	−150.5 (6)	S2—C22—C23—N2	0.0 (9)
Cl1—W1—N2—C23	118.3 (6)	S2—C22—C23—C24	−179.1 (6)
Cl2—W1—N2—C23	−73.1 (6)	C21—N2—C23—C22	0.6 (9)
C13—N1—C11—C10	176.7 (7)	W1—N2—C23—C22	−179.1 (5)
W1—N1—C11—C10	−7.8 (10)	C21—N2—C23—C24	179.7 (7)
C13—N1—C11—S1	−0.7 (8)	W1—N2—C23—C24	0.0 (10)

### Hydrogen-bond geometry (Å, °)

**Table:**

*D*—H···*A*	*D*—H	H···*A*	*D*···*A*	*D*—H···*A*
C10—H10A···O2^i^	0.99	2.38	3.28 (1)	151.

Symmetry codes: (i) *x*+1, −*y*+1/2, *z*+1/2.
